# A retrospective dosimetry study of intensity-modulated radiotherapy for nasopharyngeal carcinoma: radiation-induced brainstem injury and dose-volume analysis

**DOI:** 10.1186/s13014-018-1105-z

**Published:** 2018-10-03

**Authors:** Cheng-Yun Yao, Guo-Ren Zhou, Li-Jun Wang, Jian-Hua Xu, Jin-jun Ye, Lan-Fang Zhang, Xia He, Zhen-Zhang Chen, Sheng-Fu Huang

**Affiliations:** 1 Department of Radiation Oncology, Jiangsu Cancer Hospital & Jiangsu Institute of Cancer Research & The Affiliated Cancer Hospital of Nanjing Medical University, 42 Baiziting Road, Nanjing, 210009 People’s Republic of China; 2 Department of Medical Oncology, Jiangsu Cancer Hospital & Jiangsu Institute of Cancer Research & The Affiliated Cancer Hospital of Nanjing Medical University, 42 Baiziting Road, Nanjing, 210009 People’s Republic of China; 3 Department of Imaging, Jiangsu Cancer Hospital & Jiangsu Institute of Cancer Research & The Affiliated Cancer Hospital of Nanjing Medical University, 42 Baiziting Road, Nanjing, 210009 People’s Republic of China

**Keywords:** Nasopharyngeal carcinoma, Intensity-modulated radiotherapy, Brainstem, Radiation injury

## Abstract

**Background:**

Radiation therapy is the standard radical treatment for nasopharyngeal carcinoma (NPC) but also causes transient as well as long-term complications. Patients who develop severe radiation-induced brainstem injuries have a poor prognosis due to the lack of effective medical therapies. However, the relationship between brainstem injury and radiation volume dose is unknown. In this study, we found that radiation-induced brainstem injury was significantly associated with brainstem dose per unit volume.

**Methods:**

A retrospective analysis was performed on a consecutive cohort of 327 patients with NPC receiving IMRT from May 2005 to December 2014. Dose-volume data and long-term outcome were analyzed.

**Results:**

The median follow-up duration was 56 months (range, 3–141 months), and six with T_4_ and two with T_3_ patients had radiation-induced brainstem injuries. The 3-year and 5-year incidences were 2.2% and 2.8%, respectively. The latency period of brainstem injury ranged from 9 to 58 months, with a median period of 21 months. The Cox regression analysis showed that brainstem radiation toxicity was associated with the T_4_ stage, D_2%_ of gross tumor volume of nasopharyngeal primary lesions and their direct extensions (GTVnx), D_max_ (the maximum point dose), D_1%_, D_0.1cc_ (the top dose delivered to a 0.1-ml volume), and D_1cc_ (the top dose delivered to a 1-ml volume) of the brainstem (*p* < 0.05). Receiver operating characteristic (ROC) curves showed that GTVnx D_2%_ and the D_max_, D_1%_, D_0.1cc_, and D_1cc_ of the brainstem were significant predictors of brainstem injury. The area under the ROC curve for these five parameters was 0.724, 0.813, 0.818, 0.818, and 0.798, respectively (*p* < 0.001), and the cutoff points 77.26 Gy, 67.85 Gy, 60.13 Gy, 60.75 Gy, and 54.58 Gy, respectively, were deemed as the radiation dose limit.

**Conclusions:**

Radiotherapy-induced brainstem injury was uncommon in patients with NPC who received definitive IMRT. Multiple dose-volume data may be the dose tolerance of radiation-induced brainstem injury.

## Background

Nasopharyngeal carcinoma (NPC) is rather common among Asians, especially the Southern Chinese [1]. Radiation therapy is the standard radical treatment for NPC but also causes transient as well as long-term complications [2]. Radiation-induced brain necrosis (RN) is one of the more severe complications and can potentially lead to cognitive dysfunction, seizure, headache, and limb paralysis. The incidence of RN has been demonstrated to directly correlate with the modality of radiation therapy, which was suggested in previous studies; there is a lower occurrence in patients treated with intensity-modulated radiotherapy (IMRT) [3]. However, there are other factors that may influence the incidence and severity of radiation-induced brainstem injury, for example, long-term close follow-up, the proper diagnostic modality, and independent image interpretation.

The authors of this study recognized the importance of those aspects and accordingly analyzed the brainstem data of NPC patients treated in our center between May 2005 and December 2014. We also try to identify the relationship between the incidence of brainstem injury and radiation dose to improve the understanding of brainstem protection.

## Methods

### Patients

Inclusion criteria were as follows: (1) histologically confirmed NPC by biopsy; (2) no evidence of distant metastasis; (3) no previous treatment for NPC; (4) no pregnancy or lactation; (5) no previous malignancy or other concomitant malignant disease; (6) performance status of 0 or 1; (7) received radical IMRT at initial diagnosis; (8) no brain bleeding history; and(9)regular close follow-up with contrasted MRI. From March 2005 to September 2014, 327 newly diagnosed, biopsy-proven, consecutive NPC patients were treated in Jiangsu Cancer Hospital. 19 patients (5.5%) did not meet the research requirements and were excluded from the study. All participants were provided their written informed treatment consent, the development of this retrospective study has been approved by hospital ethics committee and is in line with the Helsinki Declaration. and all experiments were performed in accordance with relevant guidelines and regulations. Of these, magnetic resonance imaging (MRI) data allowing brainstem evaluation after completion of IMRT were available for 327. The male/female ratio was 2.7:1, and patients ranged from 12 to 77 years old (median, 48 years old). According to the 7th edition of the AJCC/UICC staging system, 24 patients had stageIdisease, 59 stage II, 111 stage III, and 133 stage IVa. By T-stage classification, 102 patients were T1, 32 T2, 80 T3, and 113 T4. All patients underwent a series of pretreatment evaluations and examinations (including history-taking, physical examination, hematological and biochemical profiling, nasopharynx and neck contrasted MRI, thoracic-abdominal computed tomography (CT), and whole- body single photon emission CT bone scanning) to exclude those with contraindications to treatment and distant metastases. This retrospective study was approved by the ethics committee of Nanjing Medical University Cancer Center.

### IMRT and chemotherapy

Inverse IMRT treatment planning was performed on a Varian Inspiration Platform (version 10.0), using the simultaneous integrated boost technique. Gross tumor volume of nasopharyngeal primary lesions and their direct extensions (GTVnx) and positive neck lymph nodes (GTVnd) were delineated according to the recommendations of the International Commission on Radiation Units and Measurements Reports nos. 50 and 62. The clinical target volume 1 (CTV1) was defined as the GTVnx with 5–10-mm margins to encompass areas at high risk of microscopic extension and the entire nasopharyngeal mucosa plus a 5-mm depth of sub-mucosal tissue. The CTV2 was defined by addition of 3–10-mm margins to the CTV1 to include areas at low risk of microscopic extension, the level of the identified positive lymph node, and the elective cervical region. The corresponding planning target volumes (PTVs) were generated from the GTVs or CTVs plus 3-mm margins to allow for setup uncertainties. The prescribed doses were 68–75 Gy to the PTV of the GTVnx in 32–34 fractions; 64–75 Gy to the PTV of the GTVnd in 32–34 fractions; 60 Gy to the PTV of CTV1 in 32 fractions; and 50 Gy to the PTV of CTV2 in 28 fractions. All patients were given one fraction daily 5 days a week. The dose-volume-histograms (DVHs) of the organs at risk were evaluated as described in the radiation therapy oncology group (RTOG) 0225 protocol to prevent violation of the tolerance limits [4].

IMRT alone was recommended for stageIpatients and IMRT combined with concurrent platinum-based chemotherapy for stage II-IVb patients [5]. Neoadjuvant chemotherapy was prescribed for patients with bulky lesions (at the primary site or in the neck); those with residual disease after IMRT received platinum-based adjuvant chemotherapy.

### Brainstem re-contouring and DVH data collection

As the brainstem had been delineated inconsistently by different radiation oncologists during original IMRT planning, we used a recommended method [6] to re-contour the brainstem. This allowed us to collect accurate data on the following dosimetric parameters: the mean dose (D_mean_), the maximum point dose (D_max_), D_1%_, D_0.1cc_ (the maximum dose delivered to a volume of 0.1 ml; the following seven parameters are similar), D_1cc_, D_5cc_, D_10cc_, D_15cc_, D_20cc_, D_25cc,_ and D_30cc_. In addition, clinical variables, such as age, sex, stage, GTV_nx_, and chemotherapy use, were included in this study; the anteroposterior diameter of the pons cistern was also analyzed for brainstem injury.

### Image assessment and the criteria for diagnosis and grading of radiation brainstem injury

The endpoint of analysis was the development of brainstem injury identified by MRI after irradiation. All MR images were reviewed independently by two examiners (L.F.Z. and S.F.H.) who were specialized in head-and-neck cancer. Consensus was reached by discussion if any initial disagreement was apparent. As both Quality-of-life (QoL) and brain function were of great importance, regular close assessment of brainstem function was always conducted after imaging diagnosis.

Diagnostic criteria for brainstem radiation toxicity refer to RN diagnosis. It is defined as a lesion of high signal on T2-weighted images and a lesion of enhancement on post-contrast images, particularly with “soap bubble” or “Swiss cheese” enhancement [7, 8]. All brainstem injury was graded according to the Common Terminology Criteria for Adverse Events (CTCAE) version 4.03, which was based on the clinical symptoms (Grade 1-mild or asymptomatic; Grade 2-moderate, not interfering with activities of daily living (ADLs); Grade 3-severe interference with ADLs, possible intervention; Grade 4-life-threatening or disabling, intervention indicated; and Grade 5-death).

### Follow-up and statistical methods

Follow-up included clinical assessment and MRI evaluation of the head and neck. The follow-up duration was calculated from the end of IMRT to the day of the final scan. All patients were regularly followed up every 3 months during the first year, every 3–6 months during the next 2 years, and annually thereafter. The use of MRI examination on the head and neck during follow-up was conducted well, and more close MRI evaluation of the nasopharynx and/or neck was performed for cases with suspected tumor recurrence or radiotherapy-induced complications. A total of 2943 MR images were collected during follow-up; an average of approximately nine scans were available for each patient. The latency period of radiotherapy-induced brainstem complications was measured from the time of IMRT completion to the first appearance of brainstem injury.

All statistical analyses were performed using SPSS software version 21.0 (SPSS, Chicago, IL, USA). Significant dosimetric parameters were further tested using the Cox proportional hazards model. Independent significant factors were assessed using receiver operating characteristic (ROC) curves to estimate the brainstem dose tolerance. Two-sided *p*-values≤0.05 were considered statistically significant.

## Results

### Survival and patterns of treatment failure

The median follow-up duration was 56 months (range, 3–141 months). The 3-year, 5-year, overall survival, local control rate, and free from distant metastasis survival rate were 88.5%, 78.7%, 93.4%, 91.4%, 85.0%, and 81.4%, respectively. The median time to recurrence was 54 months (range, 3–141 months), and that to development of distant metastasis was 52 months (range, 2–141 months). Seventy (21.4%) patients died during the follow-up period.

### Incidence and latency period of brainstem injury

**Fig. 1:**
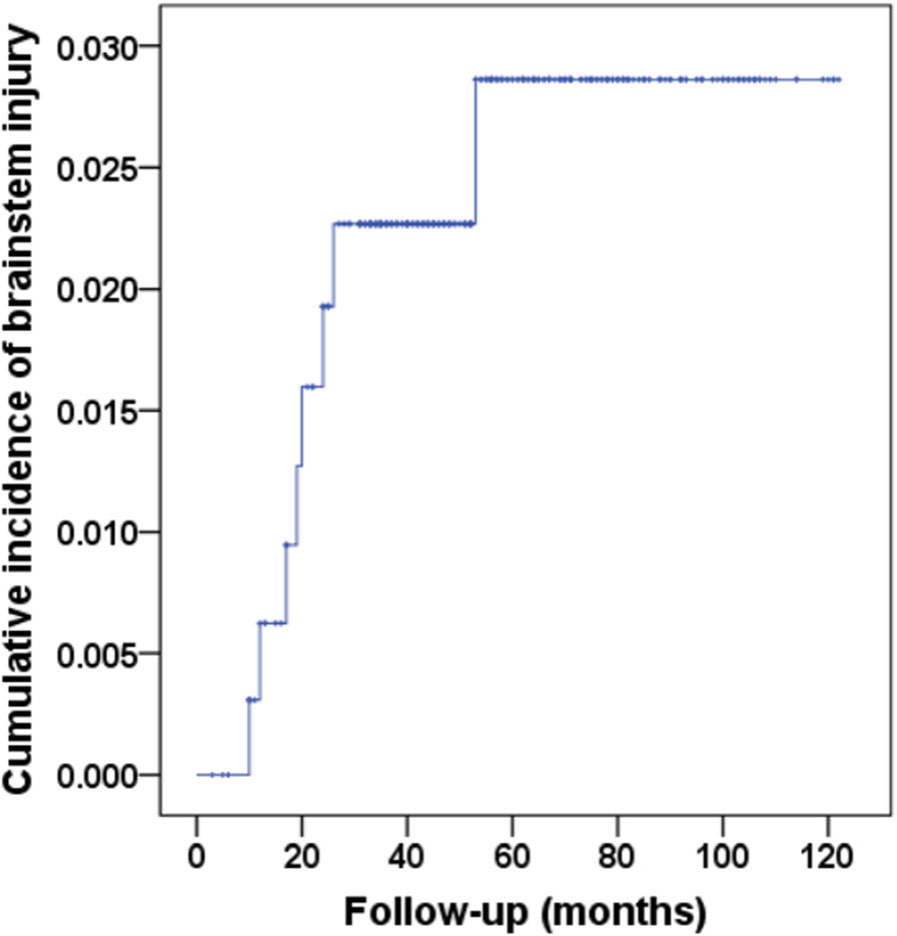
Cumulative incidence of radiation-induced brainstem injury

A total of 8 cases (8/327) developed MRI-indicated radiotherapy-induced brainstem injury. The actuarial incidence rates were 2.2% and 2.8% at 3 and 5 years, respectively; and these were 2.5% and 5.3% in T3 and T4 patients, respectively (Fig. 1). The median latency period was 21 months (range, 9–58 months).

### Clinical manifestations and characteristics of MRI

Of the eight patients who developed brainstem damage, four patients exhibited varying degrees of clinical symptoms, including lower cranial nerve palsy symptoms and fatigue; one case developed progressive limb weakness, finally becoming hemiplegic; and one patient died, with personality changes before death. The remaining 4 patients were asymptomatic. All brainstem injury patients were of stage T_3_ (2 cases) or T_4_ (6 cases) and received chemotherapy during their treatment periods. The corresponding dose distributions are shown in Table 1.

**Table 1:** Characteristics of 8 patients with brainstem injury

patient	gender	age	stage	Volume of GTVnx (cm^3^)	Lesion			
					Site	Number	size	grade
1	male	59	T4N0M0	164.6	Junctional portion of pons-oblongata	1	0.5 × 0.7 × 0.8	1
2	male	57	T4N0M0	103.9	The middle portion of pons	2	0.4 × 0.4 × 0.6 0.4 × 0.5 × 0.8	2
3	female	37	T4N2M0	102.3	The middle portion of pons	1	1.0 × 1.6 × 2.2	2
4	female	55	T3N1M0	49.3	The proximal portion of pons	1	1.2 × 1.4 × 1.5	4
5	male	53	T3N3M0	124.9	The proximal portion of pons	2	0.8 × 0.9 × 1.3 0.4 × 0.7 × 1.8	5
6	male	59	T4N1M0	51.8	The top portion of pons	1	1.3 × 1.9 × 0.9	1
7	female	42	T4N1M0	90.2	The top portion of pons	1	1.2 × 0.6 × 1.7	1
8	female	49	T4N1M0	36.5	The top portion of pons	1	1.0 × 0.7 × 0.9	2

*GTVnx* Gross tumor volume of nasopharyngeal primary lesions and their direct extensions

*Abbreviation: GTV* Gross tumor volume

**Fig. 2:**
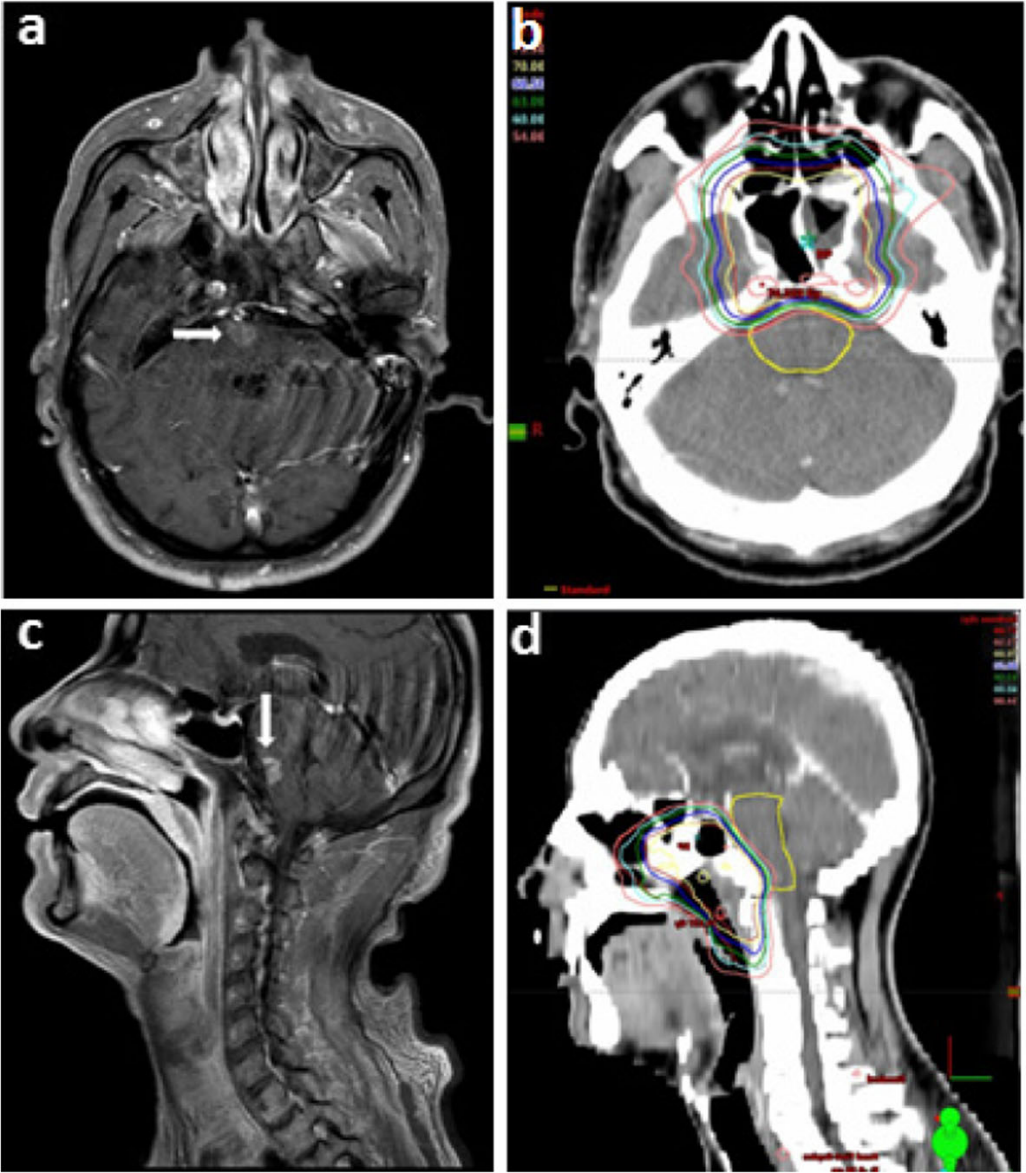
Necrosis nidus within the brainstem and the corresponding dose distribution. Contrast-enhanced lesion (white arrow) in the axial (**a**) and sagittal (**c**) view on post-contrast T1-weighted MRI images with a 53-year-old NPC patient (the 5th patient in Table 1). Corresponding isodose lines are shown in **b** and **d**, respectively

Overall, the MRI findings in the brainstem injury patients revealed a continuous spectrum of RT-associated damage. Small solid enhanced nodules were evident in four cases, while moderate and large lesions were apparent in other cases (lesions were classified as described in Table 1). The evolution of radiotherapy-induced brainstem injury was well documented; the MRI features of brainstem injury (including signal abnormality on T1 and T2 images) resolved completely in three patients following gradual improvement over two months. However, one patient developed a large contrast-enhanced lesion, with a central necrotic core in the base of the pons; this was the only patient with brainstem injury who died (Fig. 2).

### Predictive factors of radiation brainstem injury

The T category (T_4_ vs T_1–3_) as well as GTVnx D_2%_, D_max_, D_1%_, D_0.1cc_, and D_1cc_ of the brainstem were predictive factors of the radiation-induced brainstem injury in Cox regression models (*p* < 0.05) (Table 2). However, year, sex, N stage, GTVnx D_98%_, chemotherapy, anteroposterior diameter of the pons cistern, and D_mean_ were not independent risk factors of radiation-induced brainstem injury.

**Table 2:** Analysis on radiation dose and other factors affecting brainstem toxicities in Cox regression models

Factor	*p*-value	HR	95% CI for HR
T category(T_4_ vs T_1–3_)	0.029	5.94	1.2–29.43
GTVnx D_2%_	0.040	1.21	1.01–1.45
D_max_	0.003	1.15	1.05–1.26
D_1%_	0.003	1.16	1.05–1.28
D_0.1cc_	0.003	1.16	1.05–1.27
D_1cc_	0.006	1.15	1.04–1.27

*D*_*2%*_ near the maximum absorbed dose of GTV_nx_, *D*_*max*_ the maximum point dose of brainstem,

*D*_*1%*_ the dose of 1% brainstem volume, *D*_*0.1cc*_ the maximum dose of brainstem delivered to a volume of 0.1 ml, *D*_*1cc*_ the maximum dose of brainstem delivered to a volume of 1.0 ml, *GTVnx*

Gross tumor volume of nasopharyngeal primary lesions and their direct extensions

*Abbreviation: HR* hazard ratio, *CI* confidence interval, *GTV* Gross tumor volume

**Fig. 3:**
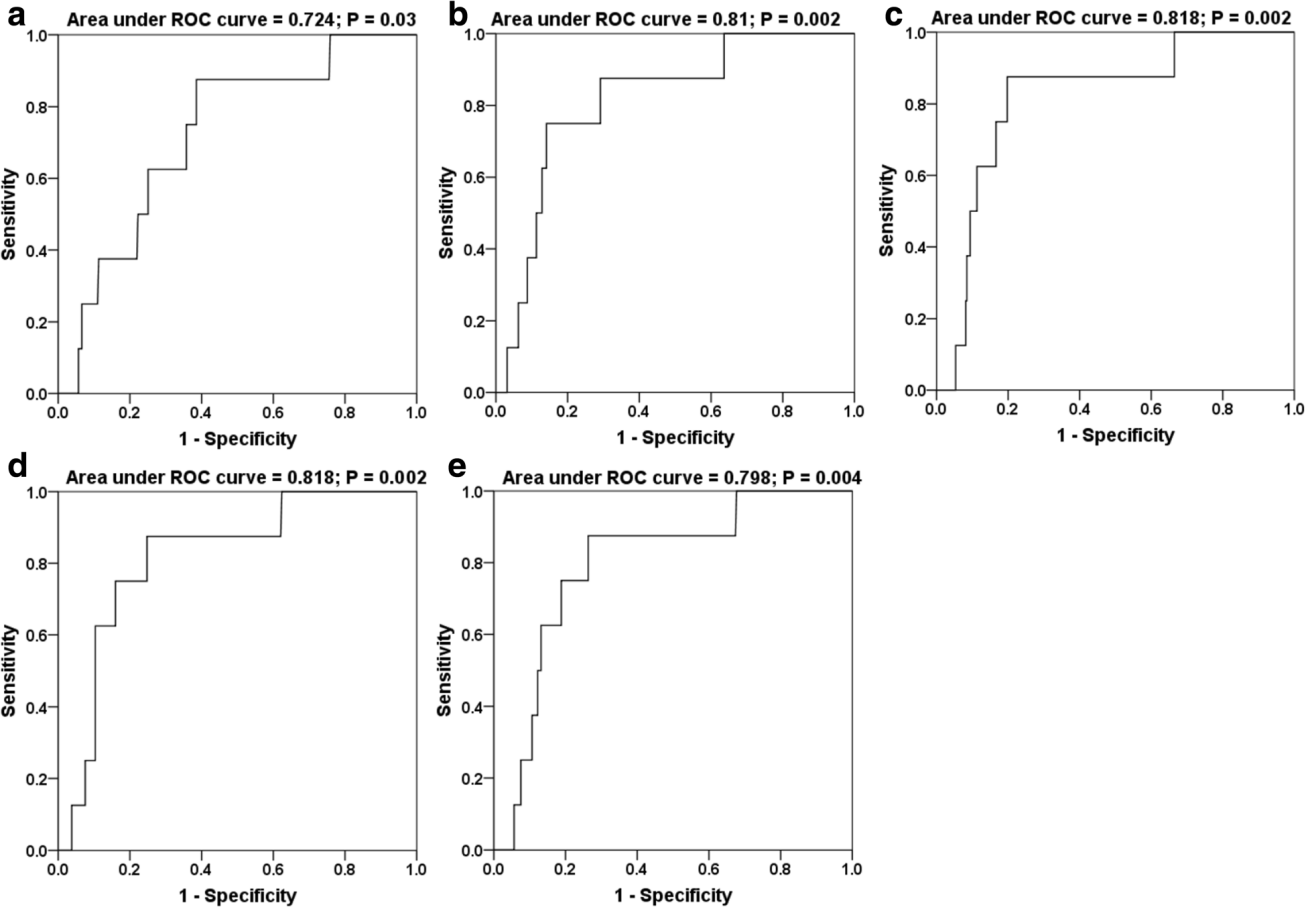
Receiver operating characteristic (ROC) curve for D2% of gross tumor volume of nasopharyngeal primary lesions and their direct extensions (GTVnx D2%) (**a**) and Dmax (**b**), D1% (**c**), D0.1 cc (**d**), and D1cc (**e**) of the brainstem, respectively. The cutoff points for the five parameters (as the brainstem dose tolerance) were determined to be 77.26 Gy, 67.85 Gy, 60.13 Gy, 60.75 Gy, and 54.58 Gy for NPC patients treated with IMRT. D2%, near the maximum absorbed dose of GTVnx; Dmax, the maximum point dose of brainstem; D1%, the dose of 1% of the brainstem volume; D0.1 cc, the maximum dose of brainstem delivered to a volume of 0.1 ml; D1cc, the maximum dose of brainstem delivered to a volume of 1.0 ml

Five qualitative variables of the predictive factors were also demonstrated by ROC curves for brainstem injury (area under the ROC curves, Table 3); the cutoff points for the dose tolerance for brainstem injury for each parameter were selected using *P* < 0.05 and Youden’s index. The parameters and cutoff values are shown in Table 4. A cumulative DVH for the dose tolerance of brainstem injury was drawn using the cutoff values (Fig. 3). The curves showed an increasing probability of brainstem injury with increasing dose; based on Fig. 3, it would be appropriate to propose a GTVnx D_2%_ of 77.26 Gy, D_max_ of 67.85 Gy, D_1%_ of 60.13 Gy, D_0.1cc_ of 60.75 Gy, and D_1cc_ of 54.58 Gy as the cutoff values for radiation-induced brainstem injury.

**Table 3:** Summary of radiation brain stem injury tolerance expressed using ROC curve

Factor	Area under ROC curve	β	*p* value	Lower limit	Upper limit	Cutoff point	sensitivity	specificity
GTVnx D_2%_	0.724	0.078	0.030	0.571	0.878	77.26	0.875	0.614
D_max_	0.813	0.067	0.002	0.682	0.945	67.85	0.750	0.859
D_1%_	0.818	0.068	0.002	0.685	0.952	60.75	0.875	0.803
D _0.1cc_	0.818	0.064	0.002	0.692	0.944	60.76	0.875	0.752
D _1cc_	0.798	0.069	0.004	0.663	0.932	54.58	0.875	0.737

*D*_*2%*_ near the maximum absorbed dose of GTVnx, *D*_*max*_ the maximum point dose of brainstem, *D*_*1%*_ the dose of 1% brainstem volume, *D*_*0.1cc*_ the maximum dose of brainstem delivered to a volume of 0.1 ml, D_1cc_ the maximum dose of brainstem delivered to a volume of 1.0 ml, *GTVnx* Gross tumor volume of nasopharyngeal primary lesions and their direct extensions

*Abbreviation: ROC* Receiver operating characteristic, *GTV* Gross tumor volume

**Table 4:** The dose of radiation brain stem injury in 8 patient

patient	GTVnx				D_1%_	D_mean_	D_max_	D_0.1cc_	D_1cc_	D_i_
	D_2%_	BED_D2%_	D_98%_	BED_D98%_						
1	77.69	94.16	72.10	87.39	64.35	49.64	68.47	65.60	62.11	61.30
2	78.60	95.26	75.01	90.91	65.20	38.04	71.59	66.96	61.46	68.60
3	82.32	99.77	73.52	89.11	65.10	44.99	69.72	65.93	59.03	64.30
4	80.66	97.76	71.23	86.33	66.44	31.75	74.07	68.64	59.25	68.20
5	73.49	89.07	68.90	83.51	60.15	36.43	63.38	60.89	56.57	58.10
6	81.79	99.13	72.70	88.11	63.73	32.40	69.41	65.79	60.00	61.73
7	78.80	95.51	72.06	87.34	61.80	36.66	67.87	63.60	54.60	60.00
8	77.32	93.71	75.25	91.20	50.94	24.85	55.30	52.47	47.77	51.71

*D*_*2%*_ near the maximum absorbed dose of GTVnx, *D*_*98%*_ near the minimum absorbed dose of GTVnx, *D*_*1%*_ the dose of 1% brainstem volume, *D*_*mean*_ the mean dose of brainstem, *D*_*max*_ the maximum point dose of brainstem, *D*_*0.1cc*_ the maximum dose of brainstem delivered to a volume of 0.1 ml, *D*_*1cc*_ the maximum dose of brainstem delivered to a volume of 1.0 ml, *D*_*i*_ injury lesion dose of brainstem, *GTVnx* Gross tumor volume of nasopharyngeal primary lesions and their direct extensions

*Abbreviation: GTV* Gross tumor volume, *BED* Biological effective dose

## Discussion

Although radiation-induced brainstem injury is uncommon, its severity has received increasing attention. Brainstem injury patients may exhibit III-XII cranial nerve palsy as well as long-beam (cone and sensory system) and cerebellar injury symptoms. Patients have no clinical symptoms in mild cases; serious complaints vary and include limb weakness, hemiplegia, gait instability, temperature sensory disturbance, diplopia, dysarthria, tongue and facial paralysis, etc. [9]. Some patients may recover from the disease after their brainstem suffers mild radiation injury, while others may need earlier medical intervention to alleviate their symptoms. However, patients who developed severe radiation brainstem injuries have a poor prognosis due to the lack of effective medical therapies.

In our research, 193 patients (59.1%) were classified as stage T_3_ or T_4_, and 8 (2.4%) brainstem injuries occurred. Patients with brainstem injuries were stage T_3_ or T_4_ of the disease, which might have resulted from the high dose of RT to the brainstem because of large tumor invasion to the skull base or to the intracranium. Both studies [10, 11] showed that brainstem injuries are related to targets that are larger and closer to the brainstem, which is similar to our results, but there was no statistical difference between patients with T_3_ and other cases, which may be due to the low rate of brainstem injury. Moreover, lack of MRI-based planning [11], the number of surgeries, hydrocephalus, diabetes, and hypertension [11–13] also contributed to injury of the brainstem. The pons cistern is located between the brainstem and basilar clivus; its anteroposterior diameter may have an impact on the brainstem dose. Our research demonstrated that there was a great variation in the anteroposterior diameter of the pons cistern in the enrolled patients, which ranged from 1.9 mm to 13.8 mm (median, 4.96 mm). However, the Cox regression analysis revealed that it was not a risk factor of radiation-induced brainstem injury.

Compared with conventional radiotherapy, IMRT reduced the radiation dose to the brainstem, temporal lobe, and other organs at risk; as a result, the incidence of radiation brainstem injury was significantly decreased. The RTOG study 0225 reported that the D_max_ of brainstem in IMRT should not exceed 54 Gy, and for patients with locally advanced NPC, this dose recommendation seems to be conservative.

General studies were undertaken to extract brainstem tolerance data. Brainstem necrosis or MRI-based evidence of injury was reported in some studies [13–17]. Five studies used photons at conventional fractionation [15, 18–21] and treatment planning limits on the high-dose component of the brainstem dose including a V_55_ < 0.1 cc [18], D_max_ < 50 Gy [19], and D_1%_ ≤ 54 Gy [21]. Uy et al. [15] reported brainstem necrosis for 1 of 40 meningioma patients treated with serial tomotherapy; D_max_ was 55.6 Gy, and the absolute volume of the brainstem that exceeded 54 Gy (aV_54_) was 4.7 ml in the treatment plan of this patient. Among 48 patients with NPC treated with 1.2 Gy/fraction twice daily to 74.4 Gy and concomitant chemotherapy, Jian noted 3 patients with Grade 1 neurologic deficit [20]. It was also reported in 367 skull-base tumor patients treated with a combination of photon and proton conformal radiation therapy between 1974 and 1995. There were 19 late brainstem-related toxicities, including three deaths. Significant predictors of toxicity by univariate analysis were as follows: D_max_ > 64 Gy, aV_50_ > 5.9 ml, aV_55_ > 2.7 ml, and aV_60_ > 0.9 ml [13, 14]. Of 208 NPC patients with more than 5 years survival after IMRT, one patient (0.48%) with a grade 2 brainstem injury and stage T_4_ (cavernous sinus) was included in the study [22, 23]. For this patient, the highest irradiation dose of the brainstem was 54.54 Gy, with a mean dose of 28.79 Gy. It was shown that the entire brainstem may be treated to 54 Gy using conventional fractionation with acceptable risk of severe or permanent neurological effects. Smaller volumes of the brainstem (1–10 cc) may be irradiated to a maximum dose of 59 Gy with conventional dose fraction (2Gy). The risk appears to increase notably when doses exceed 64 Gy [23]. However, D_max_, D_1.0cc_, and the mean dose of brainstem toxicity outcomes have not been reported in patients with NPC receiving IMRT in long-term follow-up. There is insufficient information to determine whether there is any volume effect.

In this study, brainstem injury lesion occurred most frequently in the proximal or top portion of the pons, extending to the midbrain and medulla oblongata. It may be related to the anatomical structure of the brainstem. The pons easily accepts higher doses; in areas that are located in the posterior closest to the basilar clivus, rather than the other areas, the occurrence of radiation brainstem injury is likely greater. However, lesion sites did not all occur in the most anterior portion of the pons. Four patients’ sites were located on both sides of the pons. The dose of the lesion site in 8 cases was less than its D_max_, suggesting that the occurrence of brainstem injury may be closely related to the brainstem dose per unit volume, so D_0.1cc_ and D_1cc_ were used as indicators to evaluate brainstem injury in this study.

The cutoff point of D_max_ exceeded the organ at risk (OAR) value limited by RTOG 0225/0615 in our study. There were two explanations for this abnormality. On the one hand, it was difficult to design the radiotherapy plan for the T_3_-T_4_ stage patients who had the special morphological lesions. On the other hand, insufficient attention was paid to protecting patients’ brainstems in the early stage of IMRT. At that time, no uniform standard could be referenced to contour the brainstem, which might cause the brainstem to be exposed to higher doses of radiation.

When reviewing the 8 brainstem injuries, we found two patients with diabetes. We considered their brainstem injury might be related to diabetes because their brainstem doses were not high when compared to other cases. Diabetes will cause microvessel disorder which may aggravate radiation-induced brainstem injury. Thus we reviewed all 327 patients and found that the proportion of diabetes was 12.2%, which was consistent with the most recent national survey in 2010 reported that the rate of diabetes was 11.6% [24]. Statistical analysis was not possible due to the limited cases. In the fifth and eighth cases with diabetes, the D_max_ of brainstem injury was lower than the cutoff point. Brainstem-related toxicities may be related to the microcirculation disturbance. GTVnx D_2%_, D_max_, D_1%_, D_0.1cc_, and D_1cc_ should be strictly limited in patients with local microcirculation disorders, including diabetes, high blood pressure, immune disturbance, and vascular malformations.

For patients with locally advanced NPC, sometimes it is difficult to balance tumor control and the radiation-induced brainstem injury. In those cases, we hope that the recommended dose-volume parameters are of some assistance. When the brainstem maximum dose was limited, and the unit volume dose was also strictly controlled, radiation-induced brainstem injury was uncommon. More accurate values depend on studies with larger sample sizes and longer follow-up periods.

## Conclusions

In brief, radiation-induced brainstem injury is uncommon in patients with NPC undergoing radiation therapy. Brainstem injury was significantly associated with the radiotherapy dose per unit volume.

## Abbreviations

ADLs: Activities of daily living
CT: Computed tomography
CTCAE: Common Terminology Criteria for Adverse Events
CTV: Clinical target volume
DVHs: Dose-volume-histograms
GTV: Gross tumor volume
IMRT: Intensity-modulated radiotherapy
MRI: Magnetic resonance imaging;
NPC: Nasopharyngeal carcinoma
OAR: Organ At Risk
QoL: Quality-of-life
RN: Brain necrosis
ROC: Receiver operating characteristic
RTOG: Radiation therapy oncology group
